# Cell division promotes efficient retrotransposition in a stable L1 reporter cell line

**DOI:** 10.1186/1759-8753-4-10

**Published:** 2013-03-06

**Authors:** Yi Xie, Lajos Mates, Zoltán Ivics, Zsuzsanna Izsvák, Sandra L Martin, Wenfeng An

**Affiliations:** 1 School of Molecular Biosciences and Center for Reproductive Biology, Washington State University, Pullman, WA, 99164, USA; 2 Max Delbrück Center for Molecular Medicine, Berlin, Germany; 3 Division of Medical Biotechnology, Paul Ehrlich Institute, Langen, 63225, Germany; 4 Department of Cell and Developmental Biology, University of Colorado School of Medicine, Aurora, CO, 80045, USA; 5 Current address: Department of Genetic Medicine, Weill Cornell Medical College, New York, NY, 10021, USA; 6 Current address: Institute of Genetics, Biological Research Centre, Szeged, Hungary

**Keywords:** Cell-cycle arrest, Cell-cycle synchronization, Cell division, Dual-luciferase assay, LINE-1, Non-LTR retrotransposon, Nuclear import, Tetracycline-inducible promoter, Transcription, Stable cell line

## Abstract

**Background:**

Long interspersed element type one (L1) actively modifies the human genome by inserting new copies of itself. This process, termed retrotransposition, requires the formation of an L1 ribonucleoprotein (RNP) complex, which must enter the nucleus before retrotransposition can proceed. Thus, the nuclear import of L1 RNP presents an opportunity for cells to regulate L1 retrotransposition post-translationally. The effect of cell division on L1 retrotransposition has been investigated by two previous studies, which observed varied degrees of inhibition in retrotransposition when primary cell strains or cancer cell lines were experimentally arrested in different stages of the cell cycle. However, seemingly divergent conclusions were reached. The role of cell division on retrotransposition remains highly debated.

**Findings:**

To monitor both L1 expression and retrotransposition quantitatively, we developed a stable dual-luciferase L1 reporter cell line, in which a bi-directional tetracycline-inducible promoter drives the expression of both a firefly luciferase-tagged L1 element and a Renilla luciferase, the latter indicative of the level of promoter induction. We observed an additional 10-fold reduction in retrotransposition in cell-cycle arrested cells even after retrotransposition had been normalized to Renilla luciferase or L1 ORF1 protein levels. In synchronized cells, cells undergoing two mitoses showed 2.6-fold higher retrotransposition than those undergoing one mitosis although L1 expression was induced for the same amount of time.

**Conclusions:**

Our data provide additional support for an important role of cell division in retrotransposition and argue that restricting the accessibility of L1 RNP to nuclear DNA could be a post-translational regulatory mechanism for retrotransposition.

## Findings

Long interspersed elements type one (LINE-1; L1), the only active autonomous transposable element in the human genome, have played a major role in human genome evolution and are also responsible for an increasing number of sporadic human genetic diseases [1-3]. To make new copies, a source L1 element must successfully navigate through every stage of the retrotransposition process (that is, transcription, translation, and target-primed reverse transcription). An essential intermediate step is the formation of an L1 ribonucleoprotein (RNP) complex between L1 mRNA and proteins [4-6]. L1 RNP must enter the nucleus before a new copy is made via target-primed reverse transcription [7]. Therefore, the nuclear import of L1 RNP presents an opportunity for cells to regulate L1 retrotransposition post-translationally. As nuclear import can occur passively when nuclear envelope breaks down during cell division, the efficiency of retrotransposition is predicted to be higher in actively dividing cells. Indeed, the effect of cell division has been investigated by two previous studies, which compared the level of L1 retrotransposition in cell-cycle arrested primary cell strains and cancer cell lines [8,9]. Although both observed varied degrees of inhibition in retrotransposition when cells were experimentally arrested in different stages of the cell cycle, one study concluded that cell division was required for retrotransposition and the other determined that L1 retrotransposition could occur in non-dividing cells (Table 1). Hence, the role of cell division on retrotransposition remains highly debated to date.

**Table 1:** A comparison of the methods and findings from three studies

	**Kubo *et al*.**[8]	**Shi *et al*.**[9]	**This study**
L1 vector	Embedded in a helper-dependent adenovirus	Episomal plasmid	Embedded in an SB DNA transposon
(1) Promoter	Mouse phosphoglycerate kinase-1	A native human L1 promoter (5^′^UTR)	Bi-directional tetracycline-inducible promoter
(2) ORFs	Human L1 RP	Human L1 LRE3	Synthetic mouse L1 ORFeus
(3) Reporter	EGFP	EGFP	Fluc
Gene delivery	Adenoviral transduction	Transient transfection with nucleofector	Stably integrated by SB100X
L1 expression detection	Co-expressed β-gal	L1 RNA (RT-PCR)	Co-expressed Rluc; L1 ORF1p
Cells for cell-cycle arrest assay	Human glioma (Gli36)	Human fetal lung fibroblast (IMR-90); human cervical carcinoma (HeLa)	HeLa Tet-ORFeus stable cell line
Cell-cycle arrest experiments and observed effects on retrotransposition	(i) G0 arrest ➜ complete inhibition^a^;	(i) G1, S, G2, or M arrest ➜ strong inhibition^c^	(i) S, or S+G2/M arrest ➜ strong inhibition;
	(ii) G1/S arrest ➜ partial inhibition^b^		(ii) Cell-cycle synchronized cells ➜reduced retrotransposition if cells divided one fewer cycle
Conclusion(s) regarding to the role of cell division	L1 retrotransposition can occur in non-dividing cells	Cell division is required for L1 retrotransposition; L1 transcription is the limiting step	Cell division promotes efficient L1 retrotransposition; the inhibitory effect of cell-cycle arrest on retrotransposition cannot be explained by reduced L1 transcription alone
Role of active nuclear import	L1 RNP can be actively imported into the nucleus	Not discussed	An active nuclear import mechanism is a possible explanation for residual retrotransposition in cell-cycle arrested cells

^a^Compared with cells resuming cycling, G0 arrested Gli36 cells showed a 16-fold reduction in the fraction of GFP-positive cells (54-fold if normalized to co-expressed β-gal; see Figure five B in ref [8]).

^b^Compared with cycling cells, G1/S arrested Gli36 cells showed a three-fold reduction in the fraction of GFP-positive cells (six-fold if normalized to co-expressed β-gal; see Figure four A in ref [8]).

^c^Fold reduction in retrotransposition was not stated in the main text; up to 40-fold inhibition in retrotransposition could be discerned from raw data (see Figure four A and B in ref [9]; data were not normalized to L1 RNA levels, which were reduced by approximately 10- to 20-fold in most conditions (see Figure five B in ref [9]). However, the dynamic range of observed L1 retrotransposition activity (from 0 to 40 GFP-positive cells per 10,000 cells analyzed by flow cytometry) does not allow the authors to discern additional layers of regulation for retrotransposition.

### Development of a stable HeLa Tet-ORFeus cell line

**Figure 1:**
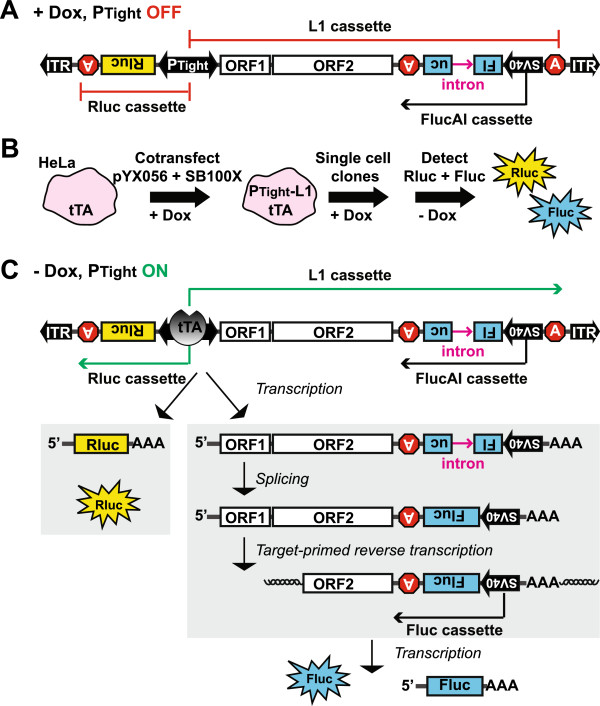
**L1 retrotransposition in a HeLa Tet-ORFeus stable cell line.** (**A**) A schematic of the bi-directional inducible L1 construct. The bi-directional tet-responsive promoter P_Tight_ drives the expression of an upstream Rluc cassette and a downstream L1 cassette. The L1 cassette features coding sequences (that is, ORF1 and ORF2) from the synthetic L1 ORFeus and an antisense-stranded FlucAI reporter cassette [11]. In the presence of doxycycline, P_Tight_ is inactive. FlucAI can be transcribed from its own SV40 promoter. However, no Fluc activity is expected because Fluc coding sequence is interrupted by an antisense intron (sense relative to the L1 cassette). (**B**) Incorporation of the L1 construct into HeLa-tTA cells. The L1 construct is terminally flanked by ITRs of the Sleeping Beauty DNA transposon (see panel **A**). To make a stable cell line, the L1 construct was co-transfected with SB100X into HeLa-tTA cells. Single cell clones were established through limiting dilution in the presence of doxycycline. Rluc and Fluc were measured after doxycycline withdrawal. (**C**) The rationale of L1 retrotransposition assay with Tet-ORFeus cells. In the absence of doxycycline, P_Tight_ is bound by tTA and activates the transcription of a Rluc mRNA and an L1 pre-mRNA. The intron is removed from L1 pre-mRNA through splicing. The mature L1 mRNA is reverse transcribed and integrated into the genome (shown as a 5^′^ truncated insertion), forming a functional Fluc cassette.

To investigate the effect of cell-cycle arrest on L1 retrotransposition, we wished to establish an assay system that meets the following criteria: (1) It must be a stable cell line with an integrated L1 reporter. Having an integrated L1 reporter eliminates variation in transfection efficiency that is inherent in transient assays. However, this requirement necessitates the use of an inducible promoter because, otherwise, L1 insertions will accumulate while the cell line is being established. (2) The promoter activity (that is, transcription) can be conveniently monitored in parallel to L1 retrotransposition. (3) Both the promoter activity and L1 retrotransposition can be measured with high sensitivity and in a wide dynamic range. Accordingly, we designed an inducible dual-luciferase L1 assay vector, pYX056 (Figure 1A; detailed in Additional file 1). The design combined a gene regulation and a gene delivery system. First, the Tet-Off Advanced Inducible Gene Expression System allows stringent control of L1 expression. The bi-directional P_Tight_ inducible promoter drives expression of Renilla luciferase (Rluc) and a hyperactive synthetic mouse L1, ORFeus [10]. The latter is tagged with a firefly luciferase/antisense intron (FlucAI) reporter cassette [11]. The bi-directional P_Tight_ promoter consists of a modified tetracycline-responsive element flanked by two minimal CMV promoters. In the presence of doxycycline, the tetracycline-controlled transactivator advanced (tTA) is complexed with doxycycline and is unable to activate P_Tight_. Upon doxycycline withdrawal, free tTA will bind to P_Tight_ and activate both L1 and Rluc transcription. Second, the non-viral two-component Sleeping Beauty (SB) system enables stable gene delivery. The L1/Rluc bi-directional expression cassette is flanked by a pair of inverted terminal repeats from SB, and can be ‘cut and pasted’ into the genome by a hyperactive SB transposase (SB100X) [12] (Figure 1B). Single cell clones were acquired by limiting dilution method and screened for the lack of Rluc expression in doxycycline-supplemented medium but high levels of Rluc expression upon doxycycline withdrawal (Figure 1C; detailed in Additional file 1).

### Control of L1 retrotransposition in HeLa Tet-ORFeus cells by doxycycline

**Figure 2:**
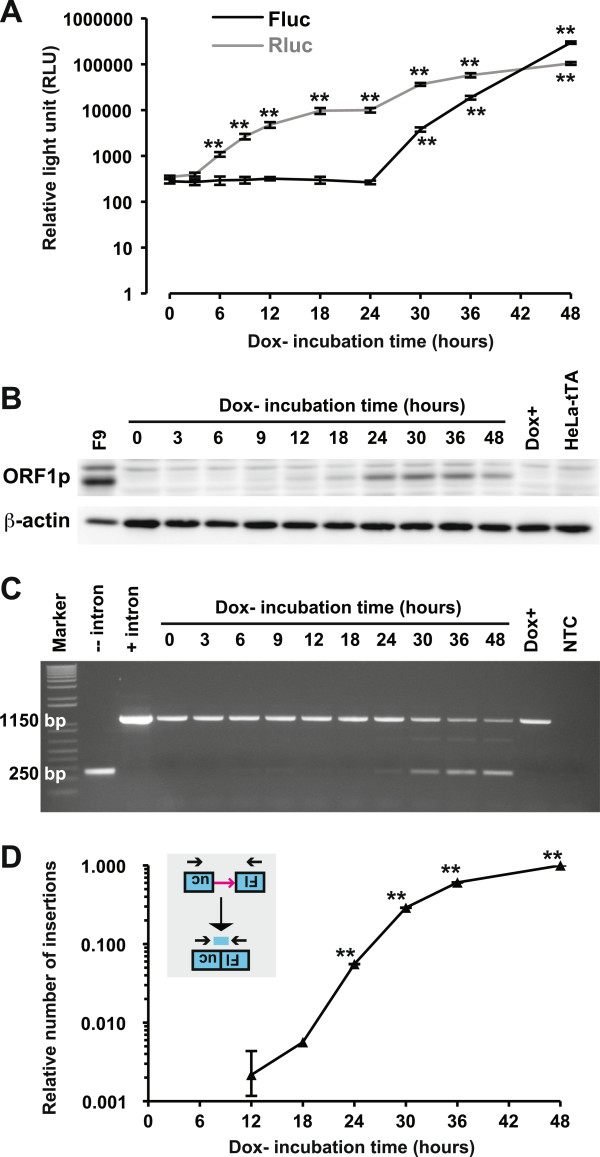
**The time course of L1 retrotransposition in HeLa Tet-ORFeus cells.** (**A**) Fluc and Rluc activities from cell lysates. Cells were seeded in 96-well plates (for luminescence) or 60 mm dishes (for protein and gDNA analyses) in the absence of doxycycline and harvested at the indicated time points. Error bars represent mean±SE (*n* = 6). All readings were compared with the 0 h control (**P <0.01). (**B**) Time-dependent increase of ORF1p expression. ORF1p and β-actin were detected by western blot. Murine embryonal carcinoma cells (F9) were used as a positive control for ORF1p. The parental HeLa-tTA cells and uninduced HeLa Tet-ORFeus cells were used as negative controls. (**C**) Confirmation of L1 retrotransposition by end-point PCR. Genomic DNA was amplified by an intron-flanking primer pair. The presence of a band of 250 bp is diagnostic for intron removal; the intron-containing donor DNA is amplified as a band of 1150 bp. NTC, no template control. Dox+, gDNA from cells cultured in the presence of doxycycline for 48 h. Fluc plasmids with or without the intron were used as controls. Molecular weight was indicated by the 1 kb Plus DNA Ladder (Invitrogen). (**D**) Quantification of L1 insertions by qPCR. The number of L1 insertions in gDNA was determined by a TaqMan-based qPCR assay. qPCR signals were normalized by setting signals from the 48 h time point to 1. The normalized signals from each time point were then compared with the 0 h time point by two-tailed Student’s *t*-test. *P* values are indicated (**P <0.01). Error bars represent mean±SE (*n* = 3).

To characterize L1 retrotransposition in HeLa Tet-ORFeus cells, we first tested the dose response by seeding cells in different concentrations of doxycycline. Both Fluc and Rluc signals were doxycycline dose-dependent (Additional file 2). Significant Fluc signals were first observed after 30 h incubation in doxycycline-free medium and subsequently increased exponentially to 460-fold above background after 48 h incubation (*P* <0.01; Figure 2A). The rapid induction of the P_Tight_ promoter via doxycycline withdrawal was demonstrated by continued increase of Rluc signals from three-fold (at 6 h) to 280-fold (at 48 h) above background (*P* <0.01; Figure 2A). To directly measure L1 expression, we quantified L1 ORF1 protein (ORF1p) by western blot (Figure 2B). L1 ORF1p signals were first observed at 9 h, peaked at 24 h, and subsequently maintained for the duration of the experiment (Figure 2B). Induction of Rluc or ORF1p was not observed in HeLa Tet-ORFeus cells cultured in 100 ng/mL of doxycycline, indicating P_Tight_ was completely suppressed. Indeed, cells maintained under 100 ng/mL of doxycycline showed no accumulation of Fluc-positive cells over 10 passages but could be robustly induced upon doxycycline withdrawal (Additional file 3). To confirm that Fluc signals were due to retrotransposition, we monitored intron removal by genomic DNA PCR as previously described [11]. Consistent with Fluc measurement, the intronless amplicon became most prominent at 30–48 h although weak amplicons could be observed in earlier time points. As a control, no intronless band was seen in HeLa Tet-ORFeus cells under 100 ng/mL of doxycycline (Figure 2C). Similar to the transient dual-luciferase assays [11], retrotransposition was inhibited by a nucleoside reverse transcriptase inhibitor in a dose-dependent manner (Additional file 4). The number of L1 insertions was further quantified by a quantitative PCR (qPCR) method as previously described [13]. Similar to transient transfection experiments [11], statistically significant signals were first detected at 24 h (normalized activity = 5.6%, *P* <0.01) (Figure 2D). It should be noted that, as opposed to antibiotic or fluorescent protein reporters, which can be used to track individual retrotransposition events, the HeLa Tet-ORFeus system measures retrotransposition from a population of cells.

### Cell-cycle arrest inhibits L1 retrotransposition

**Figure 3:**
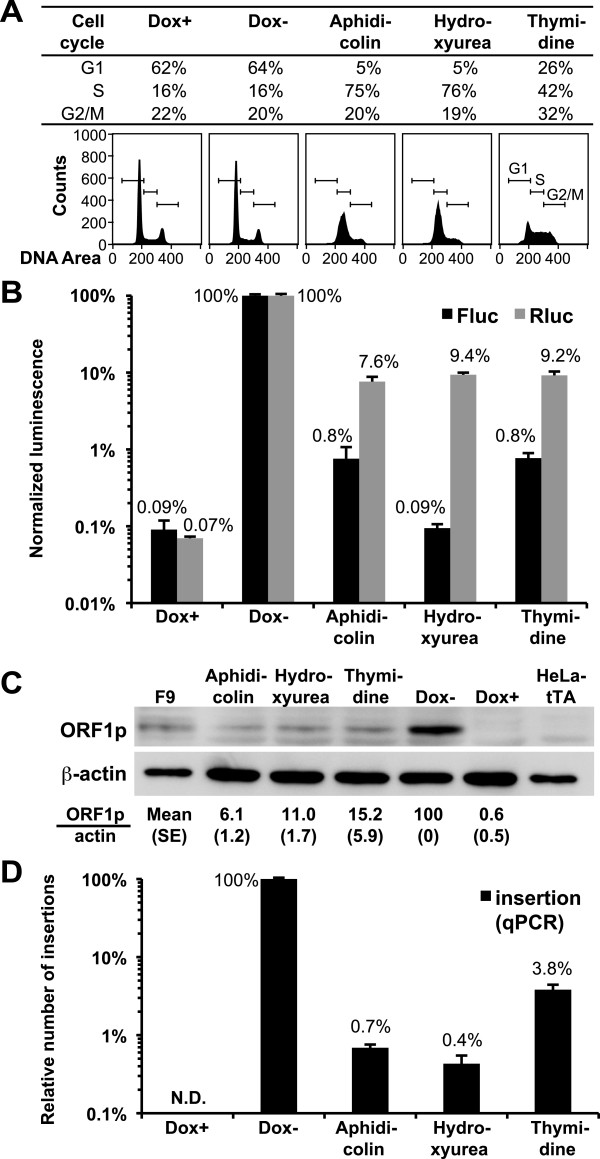
**Cell-cycle arrests inhibit L1 retrotransposition in HeLa Tet-ORFeus cells.** (**A**) Cell-cycle analysis. HeLa Tet-ORFeus cells were cultured in doxycycline-free medium (Dox-) or supplemented with 5 μg/mL aphidicolin, 75 μg/mL hydroxyurea, or 2 mM thymidine. Cells cultured in 100 ng/mL doxycycline were used as control (Dox+). The distribution of cells in different phases of the cell cycle and their corresponding DNA content histograms are shown. (**B**) Normalized Fluc and Rluc activities. HeLa Tet-ORFeus cells were treated as in panel A for 48 h. Raw luminescence readings were normalized by cell viability first and then to those from Dox- cells (Fluc_mean_ = 425,000 and Rluc_mean_ = 314,000). Error bars represent mean±SE (*n*=6). Statistical analyses are presented in Additional file 5. (**C**) The effect of cell-cycle arrest on ORF1p expression. Representative western blots were shown for ORF1p and β-actin; quantitative data were calculated from three biological replicates and had been normalized by β-actin. F9 cells were used as a positive control for ORF1p. The parental HeLa-tTA cells and uninduced HeLa Tet-ORFeus cells (Dox+) were used as negative controls. (**D**) Quantification of L1 insertions by qPCR. The number of L1 insertions in gDNA was determined by a TaqMan-based qPCR assay. qPCR signals were normalized by setting signals from the Dox- cells to 1 (equivalent to 4.9 copies per cell as estimated from plasmid DNA dilution series). Error bars represent mean±SE (*n*=3).

To test the effect of cell-cycle arrest in HeLa Tet-ORFeus cells, cells were treated with three different inhibitors in the absence of doxycycline. Cell-cycle analysis showed that cells were arrested in S phase by aphidicolin and hydroxyurea and in S+G2/M phase by thymidine (Figure 3A). The presence or absence of doxycycline had no effect on cell-cycle status (compare Dox+ with Dox- in Figure 3A). As compared with control cycling cells (that is, Dox-), arrested cells showed 7.6% to 9.4% Rluc expression, indicating P_Tight_ was suppressed in the arrested cells (Figure 3B; *P* <0.001, detailed in Additional file 5: panel A). Indeed, western blot analyses confirmed that, as compared with the Dox- group, the level of ORF1p was reduced to 6% to 15% in the arrested cells (Figure 3C). If the frequency of retrotransposition was a simple function of L1 expression, we would expect a proportional reduction of retrotransposition in arrested cells (that is, approximately 10% of cycling cells). However, the Fluc signal in arrested cells was at most 0.8% of the Dox- group (Figure 3B; *P* <0.001, detailed in Additional file 5: panel B), indicating the presence of an additional approximate 10-fold reduction in retrotransposition that cannot be explained by the decrease in L1 expression. A potential caveat for these results is that the level of retrotransposition was indirectly measured by the expression of Fluc from integrated L1 insertions; this may cause an ascertainment bias between control and treatment groups if the inhibitors affect Fluc expression. Thus, we directly quantified retrotransposition by qPCR, a method that is independent of Fluc expression. Results from these qPCR experiments confirmed the additional reduction in retrotransposition in cell-cycle arrested cells (Figure 3D): in all three treatment groups, the magnitude of decease in qPCR signal was greater than the fold reduction in L1 expression, regardless whether L1 expression is measured as Rluc or ORF1p. On the other hand, two out of the three inhibitors displayed an inhibitory effect on Fluc expression when the Fluc data and qPCR data were compared (0.09% *versus* 0.4% for hydroxyurea treated cells and 0.8% *versus* 3.8% for thymidine treated cells, respectively; compare Figure 3B and 3D). As a result, we compared the correlation between ORF1p and qPCR data. After adjusting the decrease in ORF1p, qPCR showed additional 8.7-, 27.5-, and four-fold reductions in retrotransposition in aphidicolin, hydroxyurea, and thymidine treated cells, respectively (compare Figure 3C and 3D).

### L1 retrotransposition in synchronized HeLa Tet-ORFeus cells

**Figure 4:**
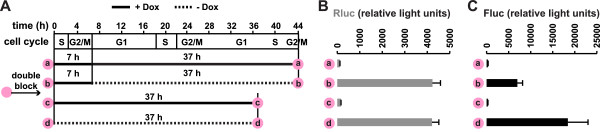
**L1 retrotransposition in synchronized HeLa Tet-ORFeus cells.** (**A**) Synchronized cells were released from double-thymidine block at time 0. Cells were incubated in the presence (solid line) or absence (dotted line) of doxycycline for varied time periods as indicated. (**B**) Rluc readouts. (**C**) Fluc readouts. Error bars represent mean±SE (*n*=6).

To exclude the possibility that the observed inhibition of L1 retrotransposition is caused by unknown side effects of inhibitors used, we wished to test the effect of cell division on L1 retrotransposition in cycling cells. To this end, we synchronized HeLa Tet-ORFeus cells by double-thymidine block and then allowed cells to enter normal cell cycling by removing thymidine from the culture medium (Figure 4A). According to cell-cycle analysis, these cells would complete two full cell cycles in 44 h (Figure 4A; Additional file 6). We compared retrotransposition under two experimental conditions (b and d in Figure 4A). L1 transcription was induced for the same amount of time in both conditions (that is, 37 h). However, the withdrawal of doxycycline was timed so that L1 expression was activated at different cell-cycle phases. As a result, when L1 expression was induced, cells in experiment b would undergo G2/M phase once whereas cells in experiment d would undergo G2/M phase twice (Figure 4A). After 37 h induction, dual-luciferase readouts were taken from both conditions. Both showed similar levels of promoter activities (Figure 4B) but the level of retrotransposition was 2.6-fold higher in experiment d than in experiment b (*P* <0.05; Figure 4C). In control experiments, we demonstrated that the difference in assay duration did not alter the assay background (Figure 4A to C; conditions a and c, where cells were released from the double-thymidine block but remained in doxycycline-supplemented medium). Thus, these data from synchronized cells further support the conclusion that cell division promotes L1 retrotransposition, and thus is a potential means of regulating L1 activity.

In summary, our data provide additional support for an important role of cell division in L1 retrotransposition and argue that restricting the accessibility of L1 RNP to nuclear DNA could be a post-translational regulatory mechanism for retrotransposition (Table 1). As compared with the two previous studies [8,9], our experimental approach has several advantages for assessing the role of cell division in retrotransposition. First, the dual-luciferase system provided an efficient means of simultaneous quantification of both L1 expression and L1 retrotransposition. Second, the use of an inducible, integrated reporter not only allowed us to avoid variation in gene transfer efficiency between experimental conditions but also to better resemble the replication cycle of endogenous L1 elements, which express from chromosomal rather than episomal DNA. Indeed, it allowed us to separate two layers of regulation in cell-cycle arrested cells: one layer is at the transcriptional level, which was highlighted by Shi *et al*. [9]; the other layer is downstream and independent of L1 transcription, as indicated by both Rluc signals and ORF1p levels (discussed below). Lastly, our inducible system permitted the comparison of retrotransposition in synchronized cell populations where the major difference was the number of mitoses completed.

Integrating our data and those of previous studies [8,9], we propose that active cell division promotes retrotransposition. All three studies showed strong inhibition of retrotransposition when cells were arrested. Shi *et al*. [9] analyzed L1 RNA levels in their assay system and attributed the inhibitory effect on retrotransposition largely to reduced L1 transcription (Table 1). The assay systems used by this study and Kubo *et al*. [8] enabled retrotransposition at larger dynamic ranges, permitting the evaluation of additional layers of regulation. In this study, after Fluc signals were normalized to the co-expressed Rluc, we observed an additional 10-fold reduction in retrotransposition in cell-cycle arrested HeLa Tet-ORFeus cells and a 2.6-fold reduction in synchronized cells undergoing one fewer round of cell division. Thus, after factoring in the effect of drug treatment on L1 expression, our data support an important role of cell division in promoting efficient L1 retrotransposition in a manner independent of L1 expression. It is noteworthy that, even when the variable infection rate was not taken into consideration, Kubo *et al*. [8] found a three-fold reduction of retrotransposition in G1/S arrested cells in addition to a 16-fold reduction of retrotransposition in G0 arrested Gli36 cells (Table 1). On the other hand, both Kubo *et al*. [8] and the current study showed substantial retrotransposition in cell-cycle arrested cells (for two of the three inhibitors tested, we observed statistically significant Fluc signals at approximately 10-fold above the assay background). Currently, we cannot exclude the possibility that the residual retrotransposition observed in arrested cell populations in both studies originates from a minor population of cycling cells. An alternative explanation for such residual retrotransposition is that L1 retrotransposition may also be facilitated by a yet uncharacterized active nuclear import mechanism (Table 1). Indeed, the control experiments performed by Kubo *et al*. [8] in G1/S arrested cells, showing differential transduction by retroviral and lentiviral vectors, support the presence of an active nuclear import mechanism for L1 retrotransposition. It is noteworthy that some non-LTR (long terminal repeat) retrotransposons have evolved active nuclear import strategies for their propagation in respective host species. A precedent of active nuclear import has been reported for the telomeric repeat-specific SART1 retrotransposon from *Bombyx mori*: its ORF1p contains functional nuclear localization signals (NLSs), which are required for active retrotransposition [14]. Thus far, no NLS has been reported in mammalian L1 proteins although both ORF1 and ORF2 proteins have highly basic regions, which is a common feature of nuclear localized proteins. Alternatively, it is possible active nuclear import is mediated by other host-derived components of L1 RNP. Recently, two poly(A) binding proteins, PABPN1 and PABPC1, were found to be associated with L1 RNP [15]. Of particular interest, PABC1 was found to be critical for RNP formation; as it can shuttle between the cytoplasm and the nucleus, it would be interesting to determine whether PABPC1 mediates RNP nuclear import [15].

A caveat shared by all three studies is that the role of cell division in retrotransposition was mainly assessed in cancerous cell lines (Table 1; but note Shi *et al*. [9] also tested normal human fetal lung fibroblasts). Additionally, two of these studies ( [8] and the current study) used non-native promoters to drive L1 expression (Table 1), which precludes the study of the endogenous transcriptional regulation of L1 with these systems. Nevertheless, a unifying view from these and other studies of L1 variants, mutations, and host factors ( [15-19] and citations therein) is that retrotransposition is not a simple function of L1 expression. By extension, the level of L1 expression cannot be equated with the frequency of retrotransposition and the evaluation of retrotransposition should take into consideration the cell-cycle status. For example, in normal individuals, endogenous L1 expression has only been confirmed at protein level in testicular and ovarian tissues ( [20-22]; reviewed in [23]). It is noteworthy that L1 ORF1p is detected in two distinct stages of male germ cell development, namely, in gonocytes (embryonic stage) and in meiotic/post-meiotic germ cells (prepuberal and through adulthood) [20-22]. However, during both stages cell division is limited: gonocytes are mitotically arrested in G0 phase [24] while spermatocytes only divide twice before becoming haploid spermatids. Similarly, in the female germline, L1 ORF1p is detected during the meiotic prophase I in embryonic oocytes [21], which are subsequently arrested in the diplotene stage of the meiotic prophase I and do not divide until puberty [25]. Therefore, both male and female germline development may have been programmed in a way that restricts excessive retrotransposition by avoiding frequent nuclear membrane breakdown when L1 is expressed. Thus, to understand the developmental timing of retrotransposition, it is imperative to measure the level of retrotransposition directly.

## Abbreviations

Fluc: Firefly luciferase; FlucAI: Fluc disrupted by an antisense intron; L1: Long interspersed element type one; NLS: Nuclear localization signal; ORF1p: Open reading frame 1 protein; qPCR: Quantitative polymerase chain reaction; Rluc: Renilla luciferase; RNP: Ribonucleoprotein; SB: Sleeping Beauty DNA transposon; tTA: Tetracycline-controlled transactivator advanced

## Competing interests

The authors declare that they have no competing financial interests.

## Authors’ contributions

YX performed the studies and drafted the manuscript. LM, ZIv, ZIz, and SM contributed reagents and provided scientific consultation. WA directed the studies and finalized the manuscript. All authors read and approved the final manuscript.

## Supplementary Material

Additional file 1: **Materials and methods.** Detailed description of materials and methods used. (PDF 129 kb) Click here for file

Additional file 2: Figure S1. Dose-dependent induction of L1 retrotransposition in HeLa Tet-ORFeus cells. HeLa Tet-ORFeus cells were seeded in 96-well plate at 3,000 cells/well and cultured in the presence of different concentrations of doxycycline. Fluc and Rluc were measured after 48 h incubation. Error bars represent mean±SE (*n*=4). At high doses in the range of 6.3 to 100 ng/mL, both Rluc and Fluc showed no deviation from background readings. Retrotransposition, as indicated by the Fluc signal, was detected in cells treated with lower doses of doxycycline. In particular, retrotransposition reached 120-fold above background under 0.8 ng/mL of doxycycline (*P* <0.001) and 3,600-fold above background in doxycycline-free medium (*P* <0.001). As expected, the level of retrotransposition was correlated with P_Tight_ promoter activity, which was measured by Rluc. At 0.8 ng/mL of doxycycline, Rluc was induced to five-fold above background (*P* <0.05); in doxycycline-free medium, Rluc was induced to 620-fold above background (*P* <0.001). It should be noted that Fluc signal had increased above background at 1.6 to 3.2 ng/mL concentrations while Rluc activity remained undetectable. This discrepancy is likely due to the known higher sensitivity of Fluc than Rluc. Thus, our data showed that L1 retrotransposition efficiency in HeLa Tet-ORFeus cells could be induced by reducing or eliminating doxycycline from the culture medium. (PDF 57 kb) Click here for file

Additional file 3: Figure S2. Induction of L1 retrotransposition in HeLa Tet-ORFeus cells after multiple passages. HeLa Tet-ORFeus cells were maintained in the presence of 100 ng/mL doxycycline and passaged in approximately every 3 days. Aliquots of cells from each of the 10 continuous passages (P0 to P9) were seeded in the presence (Dox+, shown in panel A) or absence (Dox-, shown in panel B) of 100 ng/mL doxycycline. Fluc and Rluc were measured 48 h after seeding. Note very different scales are used for the two panels. Panel A shows that Fluc and Rluc signals from uninduced cells are always below 1,000 relative light units, which represent the assay background and are comparable to readings from empty wells. Cells from most passages were seeded at the density of 3,000 to 5,000 cells/well in 96-well plates. The only exception was cells from P2, which were seeded at a much higher density (40,000 cells/well) in a 96-well plate; this suboptimal seeding density may explain the much reduced Fluc and Rluc signals in P2 cells in the absence of doxycycline (panel B). Error bars represent mean±SE (*n*=4 or 6). In summary, for cells from all passages tested, Fluc and Rluc were completely inhibited by doxycycline but were consistently induced upon doxycycline withdrawal. (PDF 67 kb) Click here for file

Additional file 4: Figure S3. Dose-dependent inhibition of L1 retrotransposition by 3TC in HeLa Tet-ORFeus cells. HeLa Tet-ORFeus cells were seeded in a 96-well plate at 3,000 cells/well and cultured in the presence of different concentrations of 2^′^,3^′^-dideoxy-3^′^-thiacytidine (3TC; 0, 0.016, 0.08, 0.4, 2, or 10 μM) and with (Dox+) or without (Dox-) 100 ng/mL doxycycline. Fluc signals were measured after 48 h incubation with Promega ONE-Glo Luciferase Assay System. Error bars represent mean±SE (*n*=8). Two-tailed Student’s *t*-test was used to compare Fluc signals from 3TC-treated cells to non-3TC-treated cells, respectively, for Dox+ and Dox- conditions; resulting *P* values are indicated (**P <0.01, ***P <0.001). (PDF 51 kb) Click here for file

Additional file 5: Figure S4. Effect of cell-cycle arrests on Rluc and Fluc activities in HeLa Tet-ORFeus cells. The underlying data are the same as in Figure 3B but Rluc and Fluc data are separately graphed to highlight the difference among experimental conditions. Raw Rluc (panel A) and Fluc (panel B) readings are shown underneath the x-axis labels. They were normalized by cell viability first and then to those from Dox- cells and plotted. Error bars represent mean±SE (*n*=6). Pairwise two-tailed Student’s *t*-test was used to compare Rluc or Fluc signals between treatment groups; resulting *P* values are indicated (*P <0.05, **P <0.01, ***P <0.001). (PDF 91 kb) Click here for file

Additional file 6: Figure S5. Cell-cycle progression after HeLa Tet-ORFeus cells released from double-thymidine block. HeLa Tet-ORFeus cells were synchronized at G1/S phase and subsequently allowed to cycle by incubating in complete medium in the absence of thymidine and doxycycline. The time of release from thymidine block was designated as time 0. Cells were collected every 4 h and subjected to cell-cycle analysis. The distribution of cell-cycle phases (G1, S, and G2/M) was plotted over time. The first column ‘C’ denotes a control population of unsynchronized cells. Note cells progressed through the first full cycle (from S, G2/M, G1 to the next S) within the first 20 h relatively synchronously but the second cycle was not as synchronous as the first cycle. (PDF 75 kb) Click here for file
