# Supplementary material for: Cell division promotes efficient retrotransposition in a stable L1 reporter cell line
Source: Mob DNA. 2013 Mar 6;4:10. doi: 10.1186/1759-8753-4-10 (PMC3607998; doi:10.1186/1759-8753-4-10)
Supplement: Additional file 1: — Materials and methods. Detailed description of materials and methods used. (PDF 129 kb) [file 1759-8753-4-10-S1.pdf]

Materials and Methods

Plasmids construction

The inducible dual-luciferase L1 assay vector pYX056 was constructed through the following subcloning steps. pTT013 is an episomal retrotransposition vector that contains a CMV promoter, ORFeus coding sequences, and the intron-disrupted NeoAI reporter [1]. pWA346 is an episomal retrotransposition vector that contains a dual CMV/CAG promoter, ORFeus coding sequences, and the intron-disrupted FlucAI reporter [2]. pYX033 contains CMV-ORFeus FlucAI; it was derived by three-way ligation of AseI/AgeI and AgeI/PspXI fragments from pTT013 and a PspXI/AseI fragment from pWA346. pCEP-Puro-CMVless is a generous gift from Yasunori Aizawa, Tokyo Institute of Technology; it was constructed by double digestion of pCEP-Puro [1] with Acc65I and BsrGI and subsequent religation. pYX020 was derived from pCEP-Puro-CMVless by filling in the EcoRI site at nucleotide position 7320 with DNA polymerase I Klenow fragment (New England Biolabs). pYX046 is an intermediate vector, which lacks a promoter but contains the ORFeus-FlucAI sequence; it was derived by ligating two EcoRI/NotI fragments: the ORFeus-FlucAI fragment of pYX033 and the CMV-less backbone of pYX020. pYX048 carries the bidirectional P_{Tight} promoter between Sleeping Beauty terminal inverted repeats (i.e. ITR-P_{Tight}-ITR); it was derived by ligating two XbaI/EcoRV fragments: the P_{Tight} promoter from pTRE-Tight-BI (Clontech Laboratories) and the SB transposon backbone from pT2BH. pYX049 contains ITR-Rluc-P_{Tight}-ITR; it was made by ligating the Rluc module as a HindIII(blunted)/PstI fragment of pTT016 [1], to a NdeI(blunted)/PstI fragment of pYX048. pTT002 is an episomal retrotransposition vector that contains CMV-L1rp-FlucAI. The L1rp-FlucAI module of pTT002 as a PvuI(blunted)/NotI fragment was ligated to ClaI(blunted)/NotI linearized pYX049, resulting pYX050. Finally, pYX056 was derived by ligating two NotI/SwaI fragments: the ORFeus-FlucAI fragment of pYX046 and the backbone of pYX050. T4 DNA ligase and restriction enzymes were purchased from New England Biolabs.

Construction of stable HeLa-Tet-ORFeus cell lines

HeLa Tet-Off Advanced cells (Clontech Laboratories) were cultured at 37°C in a humidified 5% CO_2 incubator in Dulbecco's Modified Eagle's Medium (DMEM) supplemented with 10% tetracycline-free fetal bovine serum (Clontech Laboratories), 2 mM stable dipeptide of L-alanyl-L-glutamine and 100 μg/ml G418 (Sigma-Aldrich). Cells were passaged every three days by using 0.25% porcine trypsin. To prepare for transfection, HeLa Tet-Off Advanced cells were seeded into 24-well plates at the density of 60,000 cells/well and grown up to 80–90% confluence in the presence of 100 ng/ml doxycycline (Sigma-Aldrich). Endotoxin-free plasmid DNA was prepared with anion-exchange resin columns (Qiagen) and diluted in sterile 10 mM Tris–HCl buffer. Cells were transfected with FuGENE HD transfection reagent (Roche) following the manufacturer's instructions. Briefly, each well received 200 ng of pYX056 and 20 ng of SB100X [3], 0.66 μl FuGENE HD reagent, and 25 μl GlutaMAX-I- supplemented Opti-MEM I reduced-serum medium (Invitrogen). Cells were harvested 48 h post transfection and reseeded into 96-well plates at the density of 8 cells/ml. Single cell clones were expanded, seeded into separate wells in the presence or absence of doxycycline, and measured for both Fluc and Rluc. Clones showing either leaky Rluc activity in the presence of doxycycline or no induction after withdrawal of doxycycline were discarded. Two independent HeLa Tet-ORFeus clones (No.4 and No.5) showed no luciferase activity in doxycycline-supplemented medium but high levels of induction in doxycycline-free medium. Clone No.4 was selected for subsequent

experiments. The established Tet-ORFeus cell lines are a Tet-Off system. No overt cytotoxicity was observed even at the highest dose tested (not shown); consequently, the established HeLa Tet-ORFeus cells were always maintained in 100 ng/ml doxycycline-containing medium except when they were used for retrotransposition assays. All medium components for cell culture were purchased from Hyclone unless indicated otherwise.

Inducible dual-luciferase L1 retrotransposition assay

To perform retrotransposition assays, Tet-ORFeus cells were reseeded in doxycycline-free medium and both Rluc and Fluc are simultaneously measured at defined time points. Typically, HeLa Tet-ORFeus cells were seeded into 96-well plates at the density of 3000 cells/well in doxycycline-free medium. Luciferase activity was measured 48 h after seeding. Luminescence was read on a GloMax-Multi Detection System (Promega) with the Dual-Luciferase Reporter Assay System (Promega) as previously described [2].

Cell cycle arrest, cell viability and cell cycle analysis

HeLa Tet-ORFeus cells were seeded in 96-well plates or 6-well plates at the density of 60,000 cells/ml medium in the absence of doxycycline and treated with 5 μg/ml aphidicolin, 75 μg/ml hydroxyurea, or 2 mM thymidine (Sigma-Aldrich). Cells were harvested after 48 h incubation and processed for the subsequent analysis. Cells in 96-well plates were sequentially measured for cell viability and retrotransposition (dual-luciferase measurement as above). Cell viability was measured with CellTiter-Blue Cell Viability Assay (Promega) following the manufacturer's instructions. In brief, cells were incubated with 20 μl CellTiter-Blue reagent per well for 4 h, and fluorescence was measured on a GloMax-Multi Detection System (Promega). To determine the proportion of cells in different phases of cell cycle, cells in 6-well plates were fixed in 70% ethanol overnight at -20 ºC. Fixed cells were washed with 1x phosphate buffered saline (PBS) and incubated in 500 μl 1x PBS containing 50 μg/ml propidium iodide and 100 μg/ml RNase A (Sigma-Aldrich) at room temperature for 30 minutes. DNA content was determined by FACSCalibur flow cytometry (BD Biosciences) and analyzed with FlowJo version X (Tree Star, Inc). A minimum of 10,000 cells per sample was analyzed. Additional 6-well plates were used for protein extraction and Western blotting analyses.

Western blotting analysis

Cell lysates were prepared using RIPA buffer (Pierce) mixed with 1x protease inhibitor cocktail (Sigma-Aldrich) and 1 mM PMSF (MP Biomdicals). Lysates were separated by SDS-PAGE gel and transferred onto nitrocellulose membranes (Whatman). Membranes with protein were blocked with 2% bovine serum albumin (Sigma-Aldrich) in Tris-buffered saline containing 0.1% Tween 20 (Fisher Scientific) for 1 h at room temperature, and then were incubated with primary antibodies overnight at 4 ºC. Appropriate horseradish peroxidase-conjugated secondary antibodies were added and incubated for 2 h at room temperature. The immunoreactive bands were visualized with an ECL kit (GE Healthcare). Images were taken by LAS-4000 (Fuji Medical Systems). The signals were quantified using ImageJ software (National Institute of Health). The numerical data shown in Fig.3C were normalized mean±SE values from three separate Western blotting experiments. Briefly, the intensity was measured for individual ORF1p and β-actin bands for all blots. The ORF1p signal was first normalized to the β-actin loading control for the same sample within each experiment. The calculated ORF1p/β-actin signal ratio for each sample was then normalized to the cycling cells (i.e. Dox- set to 100%) within each

experiment. Finally, the normalized ORF1p/β-actin signal ratios from all three experiments were averaged. The following antibodies were used: rabbit-anti-mouse ORF1 [5], mouse-anti-mouse β-actin (Sigma-Aldrich), anti-rabbit IgG-HRP, and anti-mouse IgG-HRP (GE Healthcare).

Genomic DNA PCR and quantitative PCR

To confirm intron removal from *de novo* L1 insertions, Fluc in genomic DNA of HeLa Tet-ORFeus cells were measured as previously described by PCR and qPCR [2].

L1 retrotransposition assay in synchronized cells

HeLa Tet-ORFeus cells were synchronized in G1/S phase by double-thymidine block with minor modifications [4]. Briefly, HeLa Tet-ORFeus cells were seeded in 6-well plates at the density of 200,000 cells per well in the presence of doxycycline and 2 mM thymidine (Sigma-Aldrich). After 18 h incubation, cells were released from thymidine treatment by reseeding into thymidine-free medium at a ratio of 1:15 into 24-well plates in the presence of doxycycline. After 9 h incubation, cells were subjected to the second round of thymidine block by adding 2mM thymidine (final concentration) to the culture medium. After 17 h incubation, cells were released from thymidine block by washing three times with 1x PBS and replacing with thymidine-free medium. The time of release from thymidine block was designated as time 0. Cells were collected at various time points and subjected for cell cycle analysis as described under the header “cell cycle arrest, cell viability and cell cycle analysis”. For retrotransposition assay in synchronized cells, cells at time 0 were released into four different conditions as shown in Figure 4. At the conclusion of the experiment, dual-luciferase measurement was taken at the end of the experiment as previously described [2].

Statistical analysis

All statistical analyses were performed with two-tailed Student’s t-test.

1. An W, Davis ES, Thompson TL, O'Donnell KA, Lee CY, Boeke JD: **Plug and play modular strategies for synthetic retrotransposons.** *Methods* 2009, **49:**227-235.
2. Xie Y, Rosser JM, Thompson TL, Boeke JD, An W: **Characterization of L1 retrotransposition with high-throughput dual-luciferase assays.** *Nucleic Acids Res* 2011, **39:**e16.
3. Mates L, Chuah MK, Belay E, Jerchow B, Manoj N, Acosta-Sanchez A, Grzela DP, Schmitt A, Becker K, Matrai J, et al: **Molecular evolution of a novel hyperactive Sleeping Beauty transposase enables robust stable gene transfer in vertebrates.** *Nat Genet* 2009, **41:**753-761.
4. Harper JV: **Synchronization of cell populations in G1/S and G2/M phases of the cell cycle.** *Methods Mol Biol* 2005, **296:**157-166.
5. Martin SL, Branciforte D: **Synchronous expression of LINE-1 RNA and protein in mouse embryonal carcinoma cells.** *Mol Cell Biol* 1993, **13:**5383-5392.
