# Supplementary figures and images for: Cell division promotes efficient retrotransposition in a stable L1 reporter cell line
Source: Mob DNA. 2013 Mar 6;4:10. doi: 10.1186/1759-8753-4-10 (PMC3607998; doi:10.1186/1759-8753-4-10)

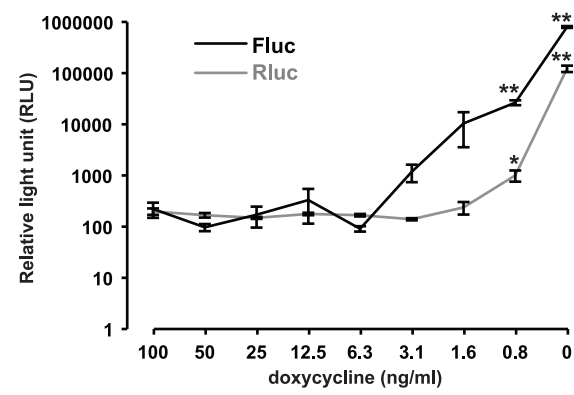

Supplement: Additional file 2: Figure S1. — Dose-dependent induction of L1 retrotransposition in HeLa Tet-ORFeus cells. HeLa Tet-ORFeus cells were seeded in 96-well plate at 3,000 cells/well and cultured in the presence of different concentrations of doxycycline. Fluc and Rluc were measured after 48 h incubation. Error bars represent mean±SE (n=4). At high doses in the range of 6.3 to 100 ng/mL, both Rluc and Fluc showed no deviation from background readings. Retrotransposition, as indicated by the Fluc signal, was detected in cells treated with lower doses of doxycycline. In particular, retrotransposition reached 120-fold above background under 0.8 ng/mL of doxycycline (P <0.001) and 3,600-fold above background in doxycycline-free medium (P <0.001). As expected, the level of retrotransposition was correlated with PTight promoter activity, which was measured by Rluc. At 0.8 ng/mL of doxycycline, Rluc was induced to five-fold above background (P <0.05); in doxycycline-free medium, Rluc was induced to 620-fold above background (P <0.001). It should be noted that Fluc signal had increased above background at 1.6 to 3.2 ng/mL concentrations while Rluc activity remained undetectable. This discrepancy is likely due to the known higher sensitivity of Fluc than Rluc. Thus, our data showed that L1 retrotransposition efficiency in HeLa Tet-ORFeus cells could be induced by reducing or eliminating doxycycline from the culture medium. (PDF 57 kb) [file 1759-8753-4-10-S2.pdf]

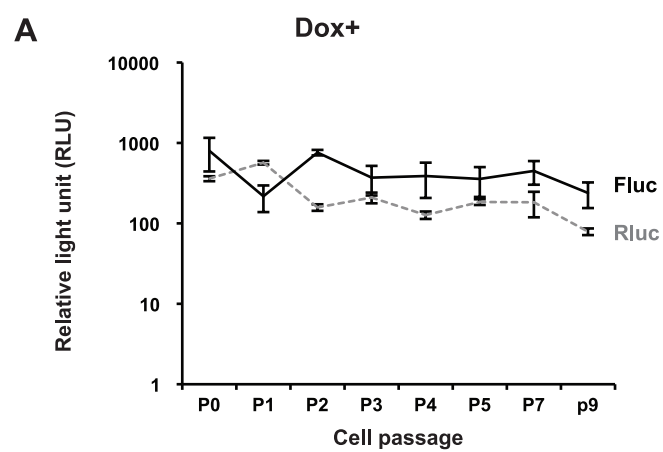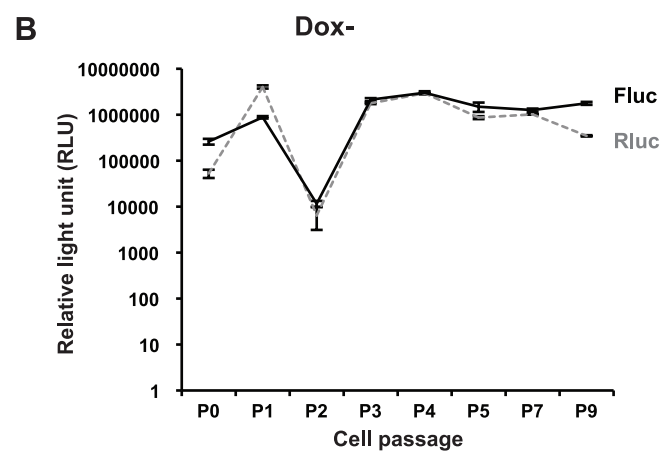

Supplement: Additional file 3: Figure S2. — Induction of L1 retrotransposition in HeLa Tet-ORFeus cells after multiple passages. HeLa Tet-ORFeus cells were maintained in the presence of 100 ng/mL doxycycline and passaged in approximately every 3 days. Aliquots of cells from each of the 10 continuous passages (P0 to P9) were seeded in the presence (Dox+, shown in panel A) or absence (Dox-, shown in panel B) of 100 ng/mL doxycycline. Fluc and Rluc were measured 48 h after seeding. Note very different scales are used for the two panels. Panel A shows that Fluc and Rluc signals from uninduced cells are always below 1,000 relative light units, which represent the assay background and are comparable to readings from empty wells. Cells from most passages were seeded at the density of 3,000 to 5,000 cells/well in 96-well plates. The only exception was cells from P2, which were seeded at a much higher density (40,000 cells/well) in a 96-well plate; this suboptimal seeding density may explain the much reduced Fluc and Rluc signals in P2 cells in the absence of doxycycline (panel B). Error bars represent mean±SE (n=4 or 6). In summary, for cells from all passages tested, Fluc and Rluc were completely inhibited by doxycycline but were consistently induced upon doxycycline withdrawal. (PDF 67 kb) [file 1759-8753-4-10-S3.pdf]

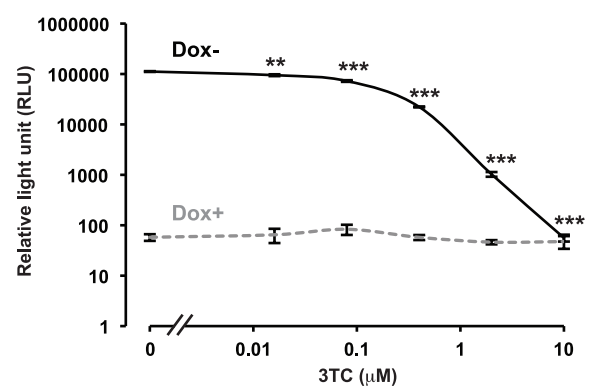

Supplement: Additional file 4: Figure S3. — Dose-dependent inhibition of L1 retrotransposition by 3TC in HeLa Tet-ORFeus cells. HeLa Tet-ORFeus cells were seeded in a 96-well plate at 3,000 cells/well and cultured in the presence of different concentrations of 2′,3′-dideoxy-3′-thiacytidine (3TC; 0, 0.016, 0.08, 0.4, 2, or 10 μM) and with (Dox+) or without (Dox-) 100 ng/mL doxycycline. Fluc signals were measured after 48 h incubation with Promega ONE-Glo Luciferase Assay System. Error bars represent mean±SE (n=8). Two-tailed Student’s t-test was used to compare Fluc signals from 3TC-treated cells to non-3TC-treated cells, respectively, for Dox+ and Dox- conditions; resulting P values are indicated (**P <0.01, ***P <0.001). (PDF 51 kb) [file 1759-8753-4-10-S4.pdf]

## A Rluc

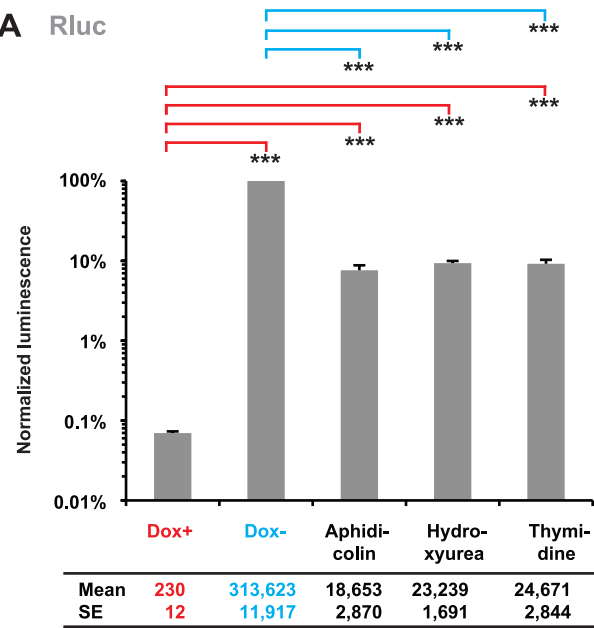

## B Fluc

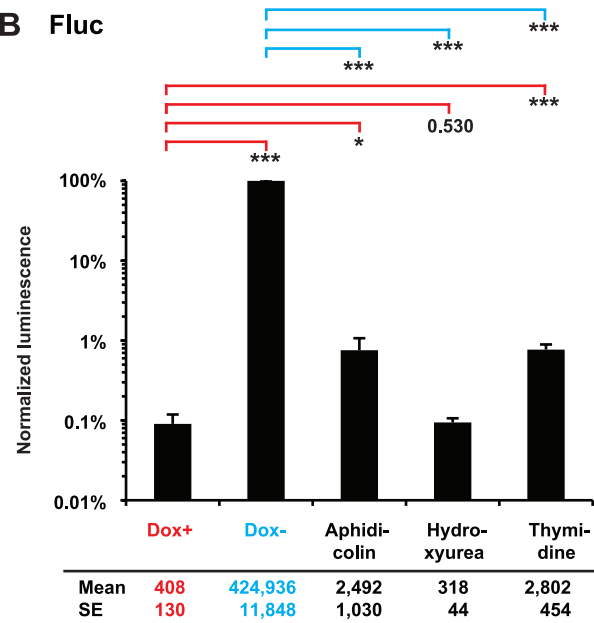

Supplement: Additional file 5: Figure S4. — Effect of cell-cycle arrests on Rluc and Fluc activities in HeLa Tet-ORFeus cells. The underlying data are the same as in Figure 3B but Rluc and Fluc data are separately graphed to highlight the difference among experimental conditions. Raw Rluc (panel A) and Fluc (panel B) readings are shown underneath the x-axis labels. They were normalized by cell viability first and then to those from Dox- cells and plotted. Error bars represent mean±SE (n=6). Pairwise two-tailed Student’s t-test was used to compare Rluc or Fluc signals between treatment groups; resulting P values are indicated (*P <0.05, **P <0.01, ***P <0.001). (PDF 91 kb) [file 1759-8753-4-10-S5.pdf]

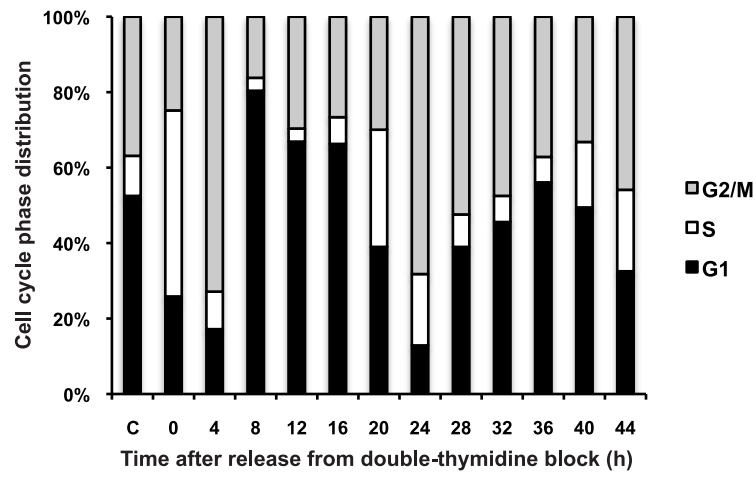

Supplement: Additional file 6: Figure S5. — Cell-cycle progression after HeLa Tet-ORFeus cells released from double-thymidine block. HeLa Tet-ORFeus cells were synchronized at G1/S phase and subsequently allowed to cycle by incubating in complete medium in the absence of thymidine and doxycycline. The time of release from thymidine block was designated as time 0. Cells were collected every 4 h and subjected to cell-cycle analysis. The distribution of cell-cycle phases (G1, S, and G2/M) was plotted over time. The first column ‘C’ denotes a control population of unsynchronized cells. Note cells progressed through the first full cycle (from S, G2/M, G1 to the next S) within the first 20 h relatively synchronously but the second cycle was not as synchronous as the first cycle. (PDF 75 kb) [file 1759-8753-4-10-S6.pdf]
